# General practitioners’ experiences of managing low back pain in primary care in Ireland: A qualitative phenomenological study

**DOI:** 10.1371/journal.pone.0339053

**Published:** 2025-12-22

**Authors:** Seán Mc Auliffe, Fiona Wilson, Geraldine Foley, Susan M. Smith, Anneka Maxwell, Rosie Daly, Kieran O’Sullivan

**Affiliations:** 1 School of Allied Health, University of Limerick, Limerick, Ireland; 2 Ageing Research Centre, University of Limerick, Limerick, Ireland; 3 Discipline of Physiotherapy, School of Medicine, Trinity College Dublin, Dublin, Ireland; 4 Discipline of Occupational Therapy, School of Medicine, Trinity College Dublin, Dublin, Ireland; 5 Discipline of Public Health and Primary Care, School of Medicine, Trinity College Dublin, Dublin, Ireland; Uttara Adhunik Medical College, BANGLADESH

## Abstract

**Background:**

Low back pain (LBP) is one of the most common symptoms presented in general practitioner (GP) consultations worldwide and is a leading cause of disability. Despite LBP clinical guidelines advocating for non-pharmacological, primary care-based management, management often diverges from recommendations.

**Objective:**

The objective of this study was to explore the lived experiences of GPs’ management of LBP in primary care, using a qualitative method.

**Methods:**

Twelve GPs across multiple Irish primary care settings were interviewed using a semi-structured guide developed in line with international LBP management guidelines. Interviews were conducted either in-person or via Zoom and audio recorded. The interviews were transcribed and the data were analysed using reflexive thematic analysis.

**Results:**

Three key themes were identified. LBP is **a common issue with increasing patient complexity**. GPs frequently encounter LBP in patients with multimorbidity, particularly in lower socioeconomic settings, complicating diagnosis and management. Secondly, GPs spoke about the **complexity of managing LBP.** Challenges identified included patient pressure for imaging, limited utility of available imaging, variability in medication prescribing, and patient misunderstandings of physiotherapy. The final theme discussed how the **current healthcare system** is not fit for purpose. Structural issues such as inequitable access to physiotherapy and the two-tiered public/private healthcare system were cited as major barriers. GPs expressed a need for better resources, education, and support to align care with clinical guidelines.

**Discussion:**

The management of LBP in Irish primary care is deeply affected by systemic inequities. Both system-level reform—particularly around equitable access to care—and the development of supportive and accessible resources are needed to empower GPs to successfully manage this significant public health issue.

## Introduction

Low back pain (LBP) is an extremely common symptom experienced by people of all ages [1,2]. It is the second most common symptom-related reason for seeking care from a general practitioner (GP)/ primary care physician [3,4]. In primary care, ‘nonspecific’ LBP — where a specific pathoanatomic diagnosis (e.g., cancer, fracture, stenosis) is not linked to the pain — accounts for the vast majority (>90%) of cases. Globally, LBP is now the number one cause of disability, surpassing mental health conditions, and respiratory and cardiovascular diseases [2,5]. From an economic perspective, the burden of LBP is estimated to be around £2·8 billion in the UK and more than AU$4·8 billion in Australia per year [6,7]. In Europe, LBP is the most common cause of medically certified sick leave and early retirement [8]. Data from Ireland in 2009 estimated the societal impact of musculoskeletal disorders, such as LBP, was €750 million each year [8], a figure that is likely to increase substantially given the increasing prevalence of LBP, and population ageing trends.

Despite the plethora of treatments and healthcare resources devoted to LBP, the back-related disability burden has increased [1,9]. Current models of care are failing, with LBP-related disability increasing by 45% from 1990 to 2010, a trend that is expected to continue over the coming years [10]. Consistent recommendations for the early management of LBP are that individuals should be provided with: self-management advice and education about the nature of LBP; reassurance that they do not have a serious disease and that symptoms will improve over time; and encouragement to avoid bed rest, stay active, and continue with usual activities, including work [11,12].

Guidelines also recommend that LBP should be managed in primary care, with specialist referral only when primary care is inadequate, and surgery only when there are specific indications [13–15]. Clinical guidelines also recommend against the use of routine imaging for patients with non-specific LBP, with imaging referral only considered if red flags are present. Despite the availability of numerous clinical guidelines, the management of LBP is often rapidly escalated to more invasive, expensive and potentially harmful interventions, which have limited evidence of effectiveness despite carrying substantial risks. Pharmacological treatments such as opioid or gabapentinoid medication are not recommended in the management of LBP. Evidence suggests benefits are small, with risks of addiction, and poorer long-term outcomes than without use [16,17]. Although guidelines discourage the use of opioids, they are widely used in many high-income countries [18,19]. Australian data show that two in every three patients who seek primary care are prescribed or recommended at least one pain medicine [20], with an increase of 39% in opioid analgesic prescriptions in the last decade [21]. Guidelines also recommend that referral for imaging (X-ray or MRI) should not be routinely used, and may even be detrimental to long-term outcomes. Despite recommendations against the routine use of imaging, 39% of patients with LBP are referred for imaging by general practitioners in Norway, 54% in the USA, and 56% in Italy [10,22,23]. It is clear there is a mismatch between what is recommended in clinical practice guidelines for LBP and what is prescribed or recommended to patients in primary care. Sub-optimal adherence to clinical practice guidelines may result in poor health outcomes for patients and unnecessary costs and resource use for the health system [24–26].

Despite the high prevalence and enormous burden of LBP on a societal and individual level in Ireland, a coordinated pathway or approach is absent in management in primary care. Healthcare reform within the Irish healthcare system (*Slaintecare)* in recent years has seen a push towards optimising community‐based care to alleviate pressure on hospital services [27]. General practice plays a central role in providing comprehensive primary healthcare across communities, including those presenting with LBP; consequently, their role in implementing effective LBP care strategies is crucial [28]. Currently, there are no national clinical LBP guidelines in Ireland, with GPs acting as the gatekeepers to elective Irish public healthcare services for the person with LBP. Consequently, an understanding of GPs’ lived experiences of managing LBP in primary care is crucial to determine the wider contextual factors at play and further explore gaps in the implementation of clinical practice recommendations to improve the management of a common and disabling complaint in primary care.

This study aimed to explore the lived experiences of GPs’ management of LBP in primary care, using a qualitative method.

## Methods

### Study design

A qualitative research design. The Consolidated Criteria for Reporting Qualitative Studies (COREQ) to guide the conducting and reporting of this study (S1 File) [29]. Ethical approval for this study was received from Trinity College Dublin School of Medicine Research Ethics Committee (***Reference number 3007***). The study was orientated in an interpretative descriptive study design. An interpretative descriptive study design is aligned with a constructivist and naturalistic orientation to inquiry [30], seeks to describe and understand a process or phenomenon (in this case, experiences of GPs’ management of LBP in primary care) through the subjective perspectives of research participants and, importantly, seeks to use this information to inform practice [31].

### Data collection

To be included in the study, participants had to be licensed members of the Irish College of General Practitioners in full- or part-time practice and who consult patients with LBP as part of their routine clinical practice. General Practitioners seeing on average fewer than two new LBP patients per week were excluded. We sought to ascertain the experiences of General Practitioners across a sociodemographic continuum in Ireland. Ireland operates a hybrid public–private healthcare system. Public services, delivered by the Health Service Executive (HSE), are primarily tax-funded and accessible at low or no cost. Due to long waiting times in the public system, a large proportion of the population hold private health insurance in 2023 to access faster care. General practitioners play a central role in the Irish healthcare system. They are typically the first point of contact for patients and act as gatekeepers to most publicly funded health services. Access to diagnostic imaging, physiotherapy, specialist consultation, and elective hospital care generally requires a GP referral. Most GPs work in community-based, privately owned practices, and are reimbursed through a mix of public funding (e.g., capitation and fee-for-service under the General Medical Services scheme) and private patient fees.

A purposive and structured variation approach was used to select the sample for this study. This method allowed us to select a sample of participants representing a wide range of expertise and experience. Depending on their geographical location, participants were offered their preferred method of performing the semi-structured interviews, either in person at their clinic or online via Zoom (Zoom Video Communications, San Jose, USA).

A member of the research team (SS), a member of the Irish College of General Practitioners, utilised her professional networks to circulate the details of the study to members. Interested GPs contacted the research team via email. In addition, snowball sampling was employed where recruited GPs were asked to forward study details to colleagues and purposive sampling were used in the later stages of recruitment to capture variation in demographic factors or key characteristics that could influence GP perspectives. Data collection period was from April – October 2024.

### Qualitative data (interview)

A semi-structured interview guide was developed to capture participants’ experiences of GPs’ management of LBP in primary care. The four-phase Interview Protocol Refinement Framework was used to develop and refine the interview guide (Castillo-Montoya, 2016). The interview guide was developed, informed by the available literature on the topic and on the clinical experience of the research group. The interview guide was piloted, and revised before study commencement by the PI with a member of the research team.

The interviews began with an introductory question about the GPs’ experience of working in primary care and the types of LBP patients they typically see in general practice. Subsequent questions explored GPs’ experiences of managing LBP in primary care. The topic guide was organised around recommended behaviours of the latest international clinical guidelines for LBP, namely: (1) referral for imaging (2) medication prescription (3) providing recommended advice on activity, and (4) providing referrals aditional treatment (Physiotherapy, Consultant in secondary care) [4,14,26,32]. The interview also explored participants’ experiences of managing LBP within their sociodemographic area, taking into consideration current health system structures and the impact of wider social determinants of health (S2 File).

All interviews were conducted by the PI (SMcA), a male physiotherapist with experience in conducting qualitative research. Given the semi-structured nature of the interview guide, the interviews adopted a flexible conversational style to facilitate participants in discussing their perspectives freely and in-depth. The capacity to probe beyond the specific questions of the interview guide assisted the PI in further exploring participants’ unique personal experiences, practices, and preferences, which shaped their perspective on managing LBP as GPs in primary care. All participants provided verbal informed consent for the qualitative study immediately before the interview. Demographics , including gender, years of GP experience, and characteristics of their medical practice, were collected prior to the interview. Field notes were taken during interviews. Interviews were audio-recorded using a digital voice recording device (Sony IC Recorder ICD-UX300) and transcribed verbatim or by using Zoom transcription software, depending on whether the interview was online (using Zoom technology) or in-person. The decision to utilise a mixed data collection approach was a pragmatic decision made by the authors in an attempt to maximise diversity of GP recruitment from a geographical perspective. However, care was taken to ensure that a similar approach was taken regardless of the data collection method (face to face or online) in terms of protocol, introductory questions, responses etc.

At the conclusion of each interview, the interviewer debriefed the participant on the main content of the interview, and time was permitted for any additional commentary to facilitate the emergence of new unanticipated information [33]. Recruitment ceased when no new themes or variants on established themes occurred with two subsequent interviews.

### Data analysis

Data analysis was iterative, whereby data were analysed as they were generated. The data were analysed using Braun and Clarke’s reflexive thematic analysis [34]. Initially, the researchers **familiarised** themselves with the data by listening to the interviews and reading the transcripts. Initial coding of the entire transcripts (both semantic and latent) was performed independently using a line-by-line **coding** approach (SMcA, RD, AM). Members of the research team met regularly to review ongoing analysis and examine overlapping codes and patterns in the data to identify tentative or **emerging themes**. Preliminary or emerging themes were then collapsed and refined into themes and final themes through discussions with the wider research team, whose contrasting views explicitly sought to challenge interpretations and ensure that the data were consistent with the codes and themes [35].

### Research team and reflexivity

Reflexivity underpinned the research process to account for potential bias amongst the research team to account for *how* subjectivity shaped our inquiry [36,37]. Reflexivity involves recognising that researchers are part of the process of co-construction of the data.

The research team consisted of the following: SMcA, a physiotherapist/researcher specialising in LBP; SS, a general practitioner in primary care; FW, a physiotherapist/researcher specialising in LBP; GF, an expert qualitative researcher; KOS, a physiotherapist/researcher specialising in LBP; RD & AM, final year BSc Physiotherapy students. The research team’s diverse academic training and experiences set the foundations for interdisciplinary dialogue and helped to promote reflexivity throughout the project [38].

### Member checking

Member checking (also termed respondent or participant validation), was undertaken by returning the data (interview transcripts) to participants before finalising the themes to ensure that the data were an accurate representation of their experiences and/or whether the findings accurately reflected their viewpoint*.*

## Results

### Participant characteristics

We interviewed 12 Participants working in primary care in Ireland between May and September 2024. Interviews were between 30–45 minutes in duration. Most participants were males (9/12 75%). Most participants worked in suburban practices. The mean years working as a GP was 19 years. Eight of the interviews were conducted in person, and four online via Zoom. Characteristics of the included participants are described in Table 1.

**Table 1:** Demographics of General practitioners’ experiences of managing low back pain in primary care.

Name	Years working as a General Practitioner	Practice Setting	Gender
**Participant 1**	18 years	Urban	Male
**Participant 2**	34 years	Suburban	Male
**Participant 3**	20 years	Suburban	Male
**Participant 4**	17 years	Urban	Female
**Participant 5**	34 Years	Suburban	Female
**Participant 6**	30 Years	Suburban	Male
**Participant 7**	16 Years	Suburban	Female
**Participant 8**	15 Years	Suburban	Male
**Participant 9**	13 Years	Urban	Male
**Participant 10**	9 Years	Suburban	Male
**Participant 11**	18 years	Suburban	Male
**Participant 12**	40 years	Urban	Male

### General practitioners on the current barriers and facilitators to managing LBP in primary care

**Fig 1:**
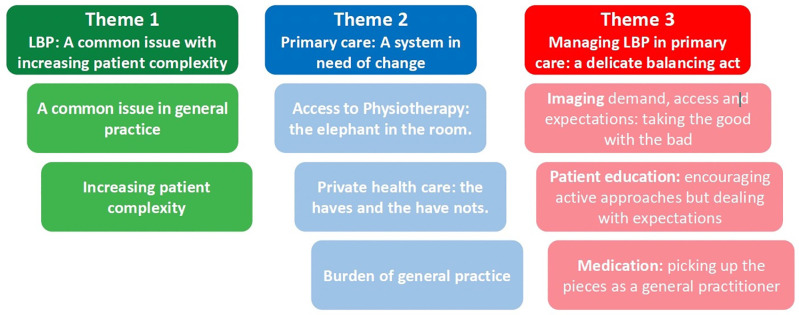
Key themes relating to general practitioners’ experiences of managing LBP.

We identified three key themes relating to Participants’ experiences of managing LBP in primary care in Ireland: 1) ***A common issue with increasing patient complexity***, 2) ***Managing LBP in primary care; a delicate balancing act***, and 3) ***A system in need of change* (Fig 1)**.

#### LBP: A common issue with increasing patient complexity.

**A common issue:** All participants reported a high prevalence of LBP in primary care. In proportional terms, several participants highlighted that LBP in primary care represents a significant proportion of their overall case load as GPs.

*“Yes, it’s very common, very, very common.*

*I’d say let’s say, say 10 to 20% of my workload would involve some form of low back pain.” GP11*

Many of the participants reported variability in the presentation of LBP in primary care, particularly between younger or middle-aged individuals and older populations. There was a sense from participants that acute LBP was often associated more with a younger or middle-aged population or those involved in some kind of physical occupation, whilst chronic or more persistent LBP was often associated with an older demographic in primary care.

*“Yeah, of the acutes. They tend to be younger, nearly all male.” GP22*

*“The percentage of people who are over 60 with lower back pain is way higher than the percentage of lower back pain patients between, say, 40 and 60.” GP11*

**Increasing patient complexity:** A common theme reported by participants was that the presentation of LBP in primary care was part of an overall increase in patient complexity in primary care, particularly in an older population. Consequently, low back pain in an older population was seen by participants as part of a whole list of numerous and interacting comorbid health complaints or multimorbidity.

*“General practice is unbelievably complex, as you know. Patients generally have a myriad of things going on. So there’s complexity and multimorbidity in every consult. I don’t think I’ve come across a clean low back pain only, because it’s mixed with obviously you know osteoporosis, obesity, you know depression, everything, everything is mixed with everything else”. GP18*

As a result, participants discussed the impact of this increased patient complexity on their ability to assess and manage LBP in primary care.

*“You know, so you have these multiple comorbidities, often obesity, lower back pain, diabetes, hypertension, depression, anxiety, and it becomes a bit of a quagmire to try and figure a way out of that one”. GP11*

Social determinants, commonly referred to as the conditions in which people are born, grow, live, work, and age, were also highlighted by participants as important factors for developing LBP. There was a feeling from the participants that these factors were associated with a higher prevalence of more chronic or persistent LBP, which had a knock-on effect in the subsequent management of LBP for a GP in primary care.

*“I am also in a low socio-economic area. I’m in one of the three. My area covers three of the 10 areas of most disadvantaged areas in the country, as per the public data. So there are more health conditions in these areas. There’s more ill health, you know, obesity and so on, and lack of activity, so back pain as well.” GP10*

*“I think we, the lower socio-economic groups tend to have, are more likely to end up with chronic lower back pain that becomes intractable and hard to manage. I think there’s a big issue then with the lower socio-economic groups, is, is it just feeds into that whole negativity….” GP11*

#### Managing LBP – A delicate balancing act.

**Imaging for LBP: taking the good with the bad:** The role of imaging and the challenges faced by participants in navigating the use of imaging in LBP were a strong theme amongst all participants. Although guidelines recommend against routine imaging for LBP [14], the pressure and realities of managing LBP in primary care highlighted the difficulty in aligning care with clinical guidelines. In the context of imaging, participants assessed the impact of recent changes in Ireland’s imaging referral pathway. Under the scheme, GPs can refer patients directly for tests, including X-ray and magnetic resonance imaging (MRI) at no cost to the patient, with access to the scheme available to the full adult population. Some participants felt that increased access to MRI for public patients improved their management of LBP in primary care.

*“You can get MRI scans in public patients on medical card patients for free through the HSE. Which has been a fantastic help I think in general.” GP12*

*“And access to imaging is much better now. But that has been hugely advantageous for us because you can tell people actually your MRI is okay first of all and now you need to work on this, that, (and) the other. Which is useful.” GP13*

*“The fortunate advent of us having access to MRIs in community, which has levelled the playing field a lot thankfully.” GP16*

In contrast, some participants felt that recent changes to imaging referral had been somewhat negative, with potential downstream consequences, and therefore resulted in little change in the management of LBP.

*“We’ve had, you know an unusual level of access, I’m not sure it’s contributed a whole pile. I don’t think it’s actually changed my practice in one case out of the 200 or 300 and I’d be quite happy to see the MRI gone.” GP20*

*“I would say 90% of the MRIs we ordering now are purely informational, rather than management related.” GP11*

In fact, some participants highlighted the fact that it has created more work for GPs as a result.

*“Because you end up with endless stuff you find on imaging than that. You have to explain or you have to refer them on to orthopaedics or neurosurgery. Most of it isn’t relevant.” GP17*

*“Well, I think it’s increased our workload because you have to go through it. And most people don’t understand it.” GP20*

Some participants felt that the additional resources that have been placed in increasing access to imaging would be better off in other areas of primary care.

*“Yeah, I think the money would be way better off spent with physio, getting better access. Resourcing physio in primary care.” G17*

A common issue cited by participants was that of managing and navigating patient expectations around the use of imaging for LBP, in particular the use of MRI. Participants commonly reported an increased demand or expectation for the need for imaging from patients with LBP.

*“In the patient’s mindset at the moment that if they have a pain in their back that maybe they should have an MRI of their back.” GP12*

*“A lot of these symptoms can be down to muscle spasm which we can’t see on an MRI scan. But there’s an expectation now they want to be imaged.” GP13*

Participants often challenged these beliefs around imaging with patients and attempted to explain the limitations of imaging for LBP when it was not warranted.

*“And I’ll say to them you know it’s not going to change our management. Okay, we’ll know what is and isn’t wrong but it’s not going to change our management.” GP13*

*“So, you know, I go back to basic(s) and exam(ine) them clinically and I would explain why I think or don’t think imaging is appropriate in terms of history and examination.” GP15*

However, in contrast, some participants often agreed to a patient’s request for imaging to show the patient there was no obvious serious pathology.

*“It’s as easy sometimes for me to do a referral in the full knowledge that nothing is going to happen out of it. But it allows, I always get the impression that people are happier if they’ve been referred on, you know.” GP20*

*“I’ll probably do it once and I just utilise that to tell them that there’s nothing wrong with it afterwards.” GP15*

**Medication management: A risk and reward process:** Medication prescription as a tool for managing LBP in primary care was a frequently discussed topic amongst participants. There was large variability across all of the participants interviewed in terms of the type of medication prescribed for individuals with both acute or more persistent LBP. Some Participants described ***“you tailor the medication to the severity of the pain”*** *GP11*, whilst others described varying combinations of anti-inflammatories, analgesics or relaxants.

*“Ah sure it depends, if it’s an acute back spasm you go in maybe with a short course of Valium and anti-inflammatories and painkillers.” GP17*

Participants were, however, unanimous in describing their reluctance or judicious use of prescribing opioids for LBP. The findings are comparable with international literature, highlighting the need to ensure that medicines are used effectively and safely for patients with low back pain to avoid unnecessary side effects and the risk of dependence [32].

*“No, no, we don’t give, I tend not to give, I never give Solpadol [*analgesic] *or opioids or anything like that.“ GP22*

*“So I’m alive to the fact that it’s really addictive and dangerous. So I try, we try and avoid it.” GP16*

The topic of deprescribing or weaning patients off certain medications in the context of LBP was cited by some Participants as an ongoing issue in primary care. Often Participants felt that patients received or were prescribed medication in secondary care settings such as ED departments or pain management specialists.

*“A lot of the time it’s actually withdrawing them off medications for the GP, don’t forget if they present in A&E which they often do or if they present, don’t forget now there’s all the VHI [private health insurance] and Laya [private health insurance] and all sorts of places they’re getting all sorts of different prescriptions. And a lot of the time it’s actually pulling them back from some of those.” GP20*

*“But what often happens is that when you refer them on to the pain clinics, they can come out from the pain clinic on huge doses of long-term analgesia. It’s a huge problem because it’s very easy to prescribe something for people and then actually supervise it and try to wean it, that’s really hard.” GP13*

As a result, Participants felt they were left to pick up the pieces as regards managing weaning or deprescribing medication for patients.

*“Yeah and again some of the times we don’t have control over this, they’ve got it elsewhere and we’re, they have seen that person once and then they’re back to us, you know.” GP20*

Participants expressed challenges in particular with medication management in older adults with LBP given the complexity of polypharmacy.

*“The older the person is the more likelier of them to have more conditions, more medications and then you have polypharmacies to watch out for.....it’s more complicated.” GP10*

As a result, Participants described a balancing act of attempting to provide pain relief to this older cohort with the potential consequences of side effects.

*“I’d be trying to keep it on a lower dose, maybe Ibuprofen if they’re older. Just to try and stop aggravation really of deterioration renal function. There’s a lot you see you have to think about.” GP16*

**Patient education: Encouraging active approaches but dealing with expectations:** Current guidelines highlight the need to ensure patients with LBP are provided with information about their condition and the need to provide targeted advice to increase their understanding, and address their concerns and expectations [39]. These recommendations were a strong theme expressed by participants, where education and advice were a common and important aspect of LBP care in primary care. Although there was some variability in what specific advice participants provided individuals with LBP, a consistent aspect was the advice to stay active. Participants expressed a view that individuals with LBP should adopt an active approach to their pain, often encouraging them to remain active.

*“Well I tell them not to go to bed. I tell them to ambulate as much as they can within reason.” GP13*

*“I say get out of the bed. Try to walk. You will find the first 5, 10, 20 steps really difficult, but it will actually loosen out after that, you know.” GP15*

However, this was often mixed with the ambiguity around how much or how little activity is appropriate for individuals with LBP.

*“And avoid heavy lifting and things like that. But actually, not really much else to be honest. Maybe I should but….” GP13*

*“I’d be telling them to stay, like moderate walking, moderate exercise, as long as they’re not exacerbating their pain.” GP17*

*“I’ll tell them to look you know, walk, walk, walk, try not to you know do any further damage to it.” GP18*

The topic of patient expectations was also raised by many participants in the context of physiotherapy for the management of LBP. Many participants believed the public perception of what physiotherapy for LBP involved was often at odds with a self-management and exercise-led approach for LBP.

*“I think the public perception of what a physiotherapist has to offer is wrong. I think that a patient expects to go to a physio and get like a massage. So if they go to the physio and they are given homework like a home exercise programme to do, they become disillusioned and they don’t engage in it.” GP13*

*“I recommend physio all the time. And I’m very specific about physiotherapy and I always, like I’m also very clear to them that I said you go to a physio not for them to put their hands on you. But you go to a physio for them to teach you how to heal yourself through your own movement. I say it’s really important you understand this because you know people come in they go, but sure I went to the physio and they did nothing for me.” GP16*

Participants also felt that these perceptions or patient expectations around the management of LBP were not unique in primary care and were common in other areas of clinical practice in the management of chronic diseases. Participants believed there was a need for patients with LBP to adopt a self-management approach to their pain which they felt was often difficult to convey to patients because of their behaviour and/or social circumstances.

*“But it’s no different to a lot of other things like somebody comes in with say an overactive bladder and I say to them stop smoking and stop drinking caffeine and stop drinking alcohol and you will be fine.“GP13*

*“And like I suppose, this isn’t a handout culture like. People have to do something for themselves.” GP14*

*“But I think that you take it for granted that people know what that is and they don’t, you know. A lot of people are brought up in houses where they know what exercise is they are involved in sport and involved in all these things they might be taught by their parents and colleagues and stuff. But loads of the population have not been exposed to that, loads of children have only ever seen what they do in school in physical education and a lot of the time they opt out of that.” GP13*

#### A system in need of change.

**A two-tiered health system:** Participants highlighted the fragmented nature of Ireland’s hybrid public/private health system as a significant barrier to managing LBP in primary care. Specifically, Participants referred to Ireland’s “two-tiered” health system, resulting in issues around access to public physiotherapy services and lack of public health initiatives as the main system constraints to managing LBP in primary care.

*“So if it’s a public patient, you put that in urgent referral letter, and then you wait for six to nine months, hopefully to get seen. If it’s a private patient, they’ll be seen within a couple of weeks.” GP11*

*“If anybody asks me your age younger or older, should I get health insurance I answer one thing. I say for orthopaedic related issues alone get health insurance. Because of course there’s a huge difference.......in terms of access.” GP18*

*“So accessing the pain clinic, the pain management, privately it’s going to be a lot quicker. Accessing the neurosurgeon for an opinion is going to be a lot quicker for the private patient. And they move onto the next stages much, much quicker.” GP19*

Some Participants highlighted the fact that access to private healthcare reduced the workload on their services in primary care, given the fact that patients often bypassed their GP in favour of allied health services such as physiotherapy.

*“You would probably see more public patients, I think private patients probably bypass us, go straight for physio and only come back to us then if there is an issue, if it’s not resolving or whatever.” GP17*

**Access to public physiotherapy:** One of the most common issues communicated by participants was the system constraint of lack of access to public physiotherapy services to assist Participants in managing LBP.

*“It’s access to physiotherapy. It’s access, timely access to physiotherapy.” GP18*

Participants interviewed highlighted significant wait times for public physiotherapy for patients with LBP.

*“Well physiotherapy access. Yeah for the public patients, yeah, we’re looking at 6 to 12 months wait list.” GP17*

*“Most of them, I suppose the reality here, you know in this network is that we don’t have access to a public physiotherapist and there’s a 12-month waiting list. Maybe it’s going to get shorter to 10 months. That’s not much use to people.” GP20*

*“Public (physiotherapy) really where I am is non-existent, you could say 6 to 12 months but that’s not….it’s either you pay for it or tough.” GP17*

Many participants expressed exasperation with the system and wait times, many reported it was futile in even referring patients for public physiotherapy as a result.

*“Oh it takes months, it’s, I don’t even refer them publicly anymore, it’s not a service.” GP18*

*“Yeah, so I probably don’t refer them anymore, I just tell them if you want physio you will have to go (yourself).” GP17*

*“I won’t even waste my energy to do a public referral and that’s no reflection on your colleagues in the public system. Because they have, they’re open and honest about the wait times around these things.” GP16*

*“So it looks like they’re getting less referrals and the waiting list isn’t as bad and isn’t that great. But actually it’s not; the reflection is that GPs are just giving up on it.” GP18*

Instead of timely access to physiotherapy for acute LBP to potentially prevent the transition to chronic LBP, some Participants felt that the wait times for public services contribute negatively towards more persistent LBP.

*“Yeah, and obviously somebody has an acute issue be it low back pain or any acute issue, psychologically it’s made into far greater by having to wait. And obviously the psychological effect on muscle spasm and everything worsens from then. So it’s access really.” GP14*

*“It’s a big issue. Like, and it’s, it’s so much of an issue. Like, if you’re waiting this for a physiotherapist is 12 months. Like that. Physiotherapist does not see acute back pain ever.” GP11*

As a result of these system constraints, Participants expressed frustration that they invariably engage in repeat consultations for LBP that could be addressed through access to appropriate and timely physiotherapy in the public system.

*“Most of them don’t yeah, they’re in pain and they’re coming back in, yeah we see repeat stuff that shouldn’t be coming to us, that should be dealt with.” GP17*

**Picking up the pieces:** Participants often described the burden and time pressures of general practice and often reported that they were the ones left to pick up the pieces when managing LBP in primary care.

*“Yeah, we’re the ones who are going to have to get the wrath for it.” GP20*

*“It’s like passing a tennis ball. I pass the tennis ball from me to the pain specialist, you know, but the tennis ball keeps coming back to me, yeah? And I can’t do any more, like, bar write a prescription.” GP10*

Patient expectations in terms of what a general practitioner could do to cure or fix an individual’s LBP were cited by participants as a common issue in primary care.

*“I think that people’s mindsets have shifted a little bit in terms of our society now wants everything to be fixed straight away.” GP13*

*“Patients don’t want the doctor to tell them, go away and do this, this, and this yourself. They want to be fixed.” GP10*

Time, and lack thereof was also often described by participants as one of the greatest burdens impacting their ability as Participants to manage LBP in primary care.

*“We don’t have time, so like we have a fifteen minute slot for appointments, and they may have come in with other things not just their back ache. So you are trying to relay huge amounts of information in a very short period of time.” GP13*

*“I don’t have time and I don’t think I, that’s where I need your colleagues to help.” GP16*

**Upskilling and resources for patients:** There were mixed feelings expressed by the Participants as regards the level of training and confidence they had as practitioners in managing LBP in primary care. Some Participants felt that the level of training was not sufficient to meet the demands of musculoskeletal pain in primary care. As a result, Participants felt that they were out of their scope of practice.

*“I would say there’s very little MSK, it may be changed now because it’s so long since, as I say I’m qualified twenty-four years now so the training now might be different.” GP12*

*I don’t tend to give people exercises, specific exercises. I don’t feel comfortable I suppose, yeah, probably just haven’t educated myself either.” GP17*

In contrast, many Participants reported undertaking professional development relating to musculoskeletal health which they found beneficial in their daily practice.

*“I think I manage low back pain reasonably, but then I would, because the way I’ve always done it I guess. I did do a.... I have a diploma in sports medicine.” GP15*

*“I’ve done an extra diploma. But having done the course, it actually just gives you that bit of extra confidence you know.” GP19*

However, participants were also unequivocal in highlighting the need for additional information and web resources to assist them in managing LBP in primary care.

*“I love a bit more education, actually. But then again, that’s just me, just being interested in the area that if someone comes in with neck pain and I want to give them range of motion exercise I’d love if there was a website if GPs could access.” GP10*

*“Right imagine you had a lovely patient information page, imagine that there you go. I’ll email you the link; I’ll text you the link there.” GP16*

## Discussion

### Summary of main findings

The qualitative approach applied yielded rich and diverse insights into GPs experiences of managing LBP in primary care in Ireland. The study explored GPs experiences across three broad themes. GPs highlighted that LBP is extremely common in everyday practice and is rarely isolated, often coexisting with obesity, depression, diabetes, etc., making management more difficult (Theme 1). Management of LBP in primary care varied, especially in the context of medication and the usefulness of imaging in the context of LBP. The majority of GPs encouraged active approaches to managing pain (walking, avoiding bed rest, etc), stressing the need for self-management. However, GPs reported challenges in shifting patient mindset away from passive care models (Theme 2). A lack of timely access to physiotherapy, compounded by the inequities of a two-tiered system often left GPs feeling burdened with repeated presentations and few effective supports or resources that could enhance care in primary settings (Theme 3).

***“General practice is unbelievably complex. Patients generally have a myriad of things going on.”***

Similar to many healthcare systems across Europe, GPs are the gatekeepers to healthcare services for individuals with LBP, especially those in receipt of publicly funded healthcare services. Consequently, the results of this study confirm that LBP is commonly seen in primary care across age groups in different countries [40].

An awareness of increasing patient complexity in the context of comorbidities was a strong discussion point expressed by GPs in managing people with LBP in primary care. LBP has traditionally been viewed as an independent disorder, seen as a purely anatomic-physiologic condition [41]. However, LBP is increasingly now seen as a chronic health condition, often co-existing with multiple long-term conditions or comorbidities (e.g., diabetes, hypertension, obesity) [42–44]. GPs in the study highlighted the fact that LBP commonly co-exists with a spectrum of chronic conditions, compounding the complexity of overall patient management. Evidence from both high- and low-income settings demonstrates a strong association between LBP and comorbidities such as cardiovascular disease, diabetes, hypertension, and mental health disorders [45–47]. A systematic review by Oliveira et al. [42] found that individuals with chronic musculoskeletal pain — including LBP — are nearly twice as likely to report cardiovascular conditions compared to those without such pain. Similarly, Ritzwoller et al. [44] reported that comorbidities, including depression, diabetes, and arthritis, significantly increased healthcare utilisation and costs among patients with LBP, particularly in those with repeated episodes. This concept of increasing patient complexity was also discussed by GPs in the context of patients’ social determinants, where such factors were associated with a higher prevalence of more persistent LBP which had a knock-on effect in the subsequent management of LBP in primary care. The increasing awareness of the impact of the social determinants of health on musculoskeletal conditions such as LBP was recently highlighted in a systematic review by Karran et al. [48]. The findings highlight strong evidence that social determinants of health, especially low education and low socioeconomic status are linked to worse outcomes in LBP.

***“But there’s an expectation now they want to be imaged.”***

Current evidence-based clinical practice guidelines for the management of LBP recommend against the routine use of imaging in patients presenting with LBP [14,15,32]. GPs in the current study appeared to be in broad agreement with these clinical guidelines, discussing that they try to ensure that a referral for imaging referral was not made on the first consultation. In contrast, some GPs interviewed reported that the presence of radicular symptoms or “sciatica” met the threshold for imaging referral, despite current guidelines recommending against the use of imaging for sciatica [32].

Similar to a review by Hall et al. [26], some GPs expressed a belief that an absence of serious pathology on imaging provides reassurance to some patients which was a justification to refer patients for imaging [26]. A common barrier around imaging cited by GPs was that of navigating patient expectations. GPs reported an increased demand or expectation from patients for imaging referral when managing LBP. These findings are not unique to an Irish setting. Previous research by Slade et al. [49] highlights the impact of perceived pressure from patients as a key driver of imaging for LBP in primary care. It is thought that patient beliefs and expectations around imaging may stem from a desire for pathoanatomical findings on diagnostic imaging to validate their pain experience [50–52]. Although GP in the current study discussed the potential positive benefits of imaging access in terms of providing reassurance, many GPs also spoke about the potential downstream effects of unintended consequences of imaging for LBP in primary care. Previous international research has highlighted that incidental findings on imaging may provoke worry or concern in patients from a labelling effect if they fail to understand that these anomalies are common and benign [53,54]. This viewpoint was highlighted by participants in this study, with GPs discussing how imaging referrals increased their workload by having to explain or discuss incidental findings with patients that have no bearing on their overall management.

***“Our society now wants everything to be fixed straight away.”***

While there were some discrepancies across the participants in terms of how they manage LBP in primary care, overall, GPs’ management strategies were consistent with current clinical practice guidelines [14,15]. Advice to avoid bed rest, stay active and maintain normal activities where possible, and reassurance or advice around prognosis were all commonly reported by GPs as part of their usual management of LBP in primary care. Exercise, particularly for those with more persistent LBP, was also commonly reported by GPs. Often, GPs believed there was a need for patients with LBP to adopt a self-management approach to their pain, often likening it to other areas of clinical practice in the management of chronic diseases. The National Institute for Clinical Excellence [32] (2017) and other clinical guidelines recognise the need for effective self-management for the life-long condition and highlight the role of healthcare providers. However, GPs also discussed how patient perceptions or expectations of what management for LBP involved often impacted their ability to engage in exercise or other self-management strategies. The viewpoint is not unique to an Irish setting, with research indicating that patients with LBP often hold strong beliefs favouring ***“quick fixes”*** or passive treatments—such as manual therapy, massage, and acupuncture, with these beliefs influenced by cultural norms, personal experiences, and interactions with healthcare providers [55,56].

***“It’s like passing a tennis ball.”***

GPs in Ireland face considerable challenges in meeting the growing demand for musculoskeletal care, including LBP. GPs frequently highlighted the lack of time and general burden of care and responsibility as a barrier to managing LBP in primary care. This is perhaps not surprising as previous research highlighted that Ireland has one of the lowest ratios of GPs per 10,000 population in Europe [57], with high levels of emotional exhaustion reported by GPs [58]. The complexities of the Irish healthcare system were also identified as a significant barrier by GPs. Ireland is one of the only Western European countries that does not offer universal coverage of primary care. The Irish healthcare system is a hybrid complex public-private mix where GPs are independent contractors and act as gatekeepers for most publicly funded primary and secondary care services. It is estimated that 43% of the population is entitled to free GP care funded by the Health Service Executive (Ireland’s public healthcare system), with fee-based GP care for the remainder in the form of private healthcare schemes or out-of-pocket payment [59]. GPs highlighted the fragmented nature of Ireland’s hybrid public/private health system, resulting in inequitable access to physiotherapy and other secondary care services. This results in private healthcare patients having quicker access to services to manage their LBP, with those without private insurance facing long waiting times for appropriate services. The findings are consistent with previous Irish research [60] demonstrating public physiotherapy services were characterised by a significantly higher percentage of patients with chronic LBP, significantly longer waiting times, and a higher number of treatments over a longer period for all LBP patients, in contrast to the private sector, where prompt management of acute LBP conditions over a relatively short period was the norm. This is compounded by geographical inequalities, with the Economic and Social Research Institute of Ireland highlighting substantial variations in primary, community and long-term care supply across regions in Ireland [61]. One of the most prominent themes of this study was GPs frustration at insufficient access to public physiotherapy services for people with LBP. Physiotherapy services in Ireland are indeed under-resourced relative to demand, with a national supply of physiotherapists 30% below the EU-28 average, with substantial inequalities in access across geographical regions [62]. Previous international research has demonstrated that physiotherapy delivered in primary care for musculoskeletal conditions is highly requested by patients and may result in health benefits and cost savings [63–66]. Given the central role of physiotherapy in evidence-based LBP management, this workforce shortage presents a major barrier to implementing guideline-recommended care and addressing the burden of care in primary care for GPs. This is further compounded by a projected rise in MSK-related disability in the future, which will place further pressure on physiotherapy and primary care services alike, highlighting the urgent need for investment in community-based capacity and workforce planning to support sustainable LBP care. An additional challenge in the context of managing LBP in primary care in Ireland relates to the absence of implementation of national LBP guidelines, which has a potential impact on GP decisions and patient outcomes. Currently, there are numerous international, national and local clinical practice guidelines for the diagnosis and management of low back pain [14]. These guidelines provide recommendations to guide clinical practice based upon the best available evidence about benefits and harms, combined with other relevant factors. Previous research has demonstrated that improved uptake of guidelines results in improved patient outcomes, such as recovery trajectory and need for ongoing care [67,68]. Although a *National Integrated Low Back Pain Pathway* has been approved in Ireland in recent years, its implementation and adoption in primary care have not been demonstrated, which may be impacting a coordinated approach to how GPs manage LBP.

### Strengths and limitations

One of the limitations of the current study may relate to the generalisability of the findings in the context of the setting. All participants were GPs in the Republic of Ireland. In addition, the unique hybrid complex public-private mix of the Irish healthcare system in primary care may mean the generalisability of the findings is limited in other international health systems and settings. Despite this, the views expressed by many of the participants aligned with previous research on the challenges of managing LBP in primary care. The authors acknowledge the variability in data collection methods may be seen as a potential limitation. A combination of face to face interviews and online technology (e.g., Zoom) was utilised during the data collection. This was a pragmatic decision in an attempt to ensure that an appropriate geographical mix of GPs by location in Ireland. However, we acknowledge this may be seen as source of potential bias. Another limitation of the current study may relate to the fact that all of the interviews were undertaken and coded by the lead author (SMcA), a physiotherapist, who may have a certain bias towards the management of LBP and the role of physiotherapy. It is possible that GPs participating in the study were aware of the lead researcher’s professional background and felt the need to highlight the role of physiotherapy. A further limitation may relate to participant diversity within the study. The majority of participants were practising in a suburban setting. This reflects the nature of general practice in Ireland, where declining population levels in rural areas have also coincided with declining levels of GPs in rural areas. In addition, although every effort was made to include an equal sample of male and female participants, the majority of participants in the study were male (75%), which is a potential limitation of the current study that should be acknowledged.

The strength of the study is underpinned by the various strategies employed to ensure methodological rigour, including researcher responsiveness, verification of data, methodological coherence and member checking (S3 and S4 Files). The purposive sampling approach was also a strength, as it ensured that participants had a wide range of practice experience and cared for populations from varied socio-economic strata. Furthermore, the authorship team is diverse, including other health care professionals who provided insight and contributed to the study.

## Conclusion

From the perspective of GPs in primary care in Ireland, the management of LBP in Irish primary care is deeply affected by systemic inequities, increasing patient complexity, limited access to physiotherapy, and structural pressures within the healthcare system. These challenges can lead to avoidable repeat consultations, delayed interventions, and growing frustration among GPs. Addressing these issues requires both system-level reform—particularly around equitable access to care—and the development of supportive, accessible resources to empower GPs and patients alike.

## Supporting information

S1 File COREQ. (PDF)

S2 File Interview guide. (PDF)

S3 File Audit trail. (PDF)

S4 File Codebook. (XLSX)
