# Supplementary material for: General practitioners’ experiences of managing low back pain in primary care in Ireland: A qualitative phenomenological study
Source: PLoS One. 2025 Dec 22;20(12):e0339053. doi: 10.1371/journal.pone.0339053 (PMC12721527; doi:10.1371/journal.pone.0339053)
Supplement: S2 File — (PDF) [file pone.0339053.s002.pdf]

S2 Appendix: interview guide

Firstly, I'd like to thank you again for taking part in this study. Before starting, I would just like to inform you that this interview is being recorded to ensure that no important information is missed. Are you happy for this to be recorded?

We are conducting this interview to explore your experiences of managing low back pain as a general practitioner working in primary care.

In this regard, I have a number of questions I would like to ask you. If you are unsure about any question or do not wish to answer, please do not worry, just let me know. Also, please be aware that there are no right or wrong answers as responses are very individual, and therefore answers are expected to be varied. Some questions at the beginning of the interview will only require a short answer about your general background, and as we progress, I will sit quietly more often and not interrupt to allow you to expand on any answers in relation to your experiences managing low back pain.

If you feel uncomfortable at any stage and feel you want to withdraw then please know you can do so at any time, without the need to give a reason. Please be aware that participating in this study will have no influence on team selection processes, as this will be determined by rugby performances in training and competition. Finally, all responses will be kept completely confidential to the research team so we would be very grateful if you could answer as honestly as possible.

This interview will take around 45 to 60 minutes. Please tell me if you feel uncomfortable to answer any question; we will then move on to the next question.

Do you have any questions before we start?

<u>Indicative content and questions</u>

Type of Question	Explanation of Type of Question	Example of Type of Question
**Introductory Questions**	Questions that are relatively neutral eliciting general and non-intrusive information and that are not threatening	Thank you for agreeing to take part in this research project. The purpose of this interview is to learn more about your experiences of managing low back pain in primary care as a general practitioner.<br><br>1. Firstly, can you begin by discussing if musculoskeletal pain, and in particular LBP is a prevalent condition in your daily practice.<br><br>**Prompts:**<br>- How much do you see

		- What is the profile of people who present to your GP surgery with LBP (age, sex, gender, socioeconomic, private v public)<br>- What would the proportion of acute vs chronic LBP<br>- Do you often see people with CLBP and other health/comorbid health conditions
**Key Questions**	Questions that are most related to the research questions and purpose of the study	The next questions aim to determine your current approach to managing LBP in primary care.<br><br>1. What is the biggest challenge for you as a GP working in primary care in relation to managing LBP in Primary care<br><br>**Prompts:**<br>- patients with mixed profiles needing a mix of physical, psych, lifestyle rehab<br><br>2. Can you discuss your rationale or circumstances around ordering imaging in LBP<br><br>**Prompts:**<br>- When do you refer someone for an MRI or xray<br>- What has the impact of the GP referral scheme been on your practice.<br><br>3. Would you mind discussing the role medication plays in managing LBP (i.e. simple analgesics or opioids)?<br><br>**Prompts:**<br>- Acute LBP – what? How long?<br>- Persistent LBP? What, how much are they involved.<br>- For those on multiple meds, how does de-prescribing work - role for GP v pharmacists v pain medicine consultant?<br><br>4. Do you ever provide advice around exercise for people with LBP?

		**Prompts:**<br>- What exactly do you advise them regarding exercise<br>- Is there anything you tell them avoid? (either an activity, or a specific exercise)<br>- Do you refer patients to Physiotherapy for LBP – Why/why not?<br><br>5. What resources - if any - do you use to guide your management, or to give to patients?'<br><br>**Prompts:**<br>- Are there any particular resources that you use with patients or to inform your care?<br>- Do you feel there is a need for the development of a resource(s) to assist GPs to manage LBP (PA Outlets – community resources…)<br><br>6. In the Irish Healthcare system patients can be public vs private. Are there any differences on how you manage these patients in the context of LBP?<br><br>**Prompts:**<br>- LBP (or pain) not being one of the chronic disease programmes<br><br>7. Finally I would like to discuss how you manage sick leave/return to work certification in the context of LB in primary care?
**Closing Questions**	Questions that are easy to answer and provide opportunity for closure	Before we conclude this interview, Is there anything else you would like to say before we finish this interview?<br><br>Thank you very much for your time.
