# Supplementary material for: General practitioners’ experiences of managing low back pain in primary care in Ireland: A qualitative phenomenological study
Source: PLoS One. 2025 Dec 22;20(12):e0339053. doi: 10.1371/journal.pone.0339053 (PMC12721527; doi:10.1371/journal.pone.0339053)
Supplement: S3 File — (PDF) [file pone.0339053.s003.pdf]

Audit Trail – Qualitative Study

Study Title:

General practitioner's experiences of managing low back pain in primary care: a qualitative study

Authors:

Seán Mc Auliffe, Fiona Wilson, Geraldine Foley, Susan Smith, Anneka Maxwell, Rosie Daly, Kieran O'Sullivan

1. Research Design

- Aim:

To qualitatively explore the lived experiences of general practitioners (GPs) managing low back pain (LBP) in Irish primary care, with a focus on barriers, enablers, and system-level challenges.

- Methodological Orientation:

Interpretive Description underpinned by a constructivist and naturalistic inquiry framework.

- Theoretical Framework:

Reflexive Thematic Analysis (Braun & Clarke, 2006).

- Ethics Approval:

Approved by Trinity College Dublin Research Ethics Committee (Reference number 3007).

- Reporting Standard:

Consolidated Criteria for Reporting Qualitative Studies (COREQ) checklist.

2. Participant Recruitment

- Sample Size:

12 general practitioners – all members of the Irish College of General Practitioners

- Sampling Strategy:

Purposive and snowball sampling through Irish College of General Practitioners networks.

- Inclusion Criteria:

Licensed GPs practicing in Ireland who regularly consult patients with low back pain

- Recruitment Timeframe:

May to September 2024.

3. Data Collection

- Method:

Semi-structured interviews guided by a protocol aligned with international LBP management guidelines.

- Interview Mode:

Conducted either face-to-face or via Zoom.

- Recording & Transcription:

Audio-recorded, transcribed verbatim (manual and Zoom-based transcription).

- Field Notes:

Taken during all interviews.

- Data Collection End Point:

Recruitment ceased when no new substantive themes or variants emerged

4. Data Management

- Anonymization:

All transcripts de-identified before analysis.

- Secure Storage:

Password-protected University of Limerick servers.

- Data Organization:

Files labeled systematically (e.g., "GP10_transcript").

5. Data Analysis

- Approach:

Reflexive Thematic Analysis using a six-phase process.

- Coding:

Initial line-by-line coding independently by three researchers (Seán Mc Auliffe, Anneka Maxwell, Rosie Daly).

- Theme Development:

Codes were collapsed and refined into 3 final themes after extensive discussion and consensus within the team.

- Sample Coding Extract:

(See attached document)

6. Trustworthiness Measures

- Reflexivity:

Reflexive memos maintained by researchers to document positionality and assumptions.

- Team Debriefing:

Regular meetings to challenge interpretations and validate coding decisions.

- Member Checking:

Participants reviewed their transcripts and validated interpretations.

- Audit Trail Documentation:

Coding frameworks, theme development logs, reflexivity memos, and meeting notes were kept systematically.

7. Outputs and Documentation

- Codebook:

Codes linked systematically to quotes, descriptions, and final themes (See attached document)

- Final Themes Identified:

1. A common issue with increasing patient complexity

2. Managing LBP: a delicate balancing act

3. A system in need of change

- Manuscript Produced:

Full manuscript drafted and structured according to COREQ guidelines.
